# DNA and Morphology Unite Two Species and 10 Million Year Old Fossils

**DOI:** 10.1371/journal.pone.0052083

**Published:** 2012-12-20

**Authors:** Simon F. K. Hills, James S. Crampton, Steven A. Trewick, Mary Morgan-Richards

**Affiliations:** 1 Ecology Group, Institute of Natural Resources, Massey University, Palmerston North, New Zealand; 2 GNS Science, Lower Hutt, New Zealand; University of Otago, New Zealand

## Abstract

Species definition and delimitation is a non-trivial problem in evolutionary biology that is particularly problematic for fossil organisms. This is especially true when considering the continuity of past and present species, because species defined in the fossil record are not necessarily equivalent to species defined in the living fauna. Correctly assigned fossil species are critical for sensitive downstream analysis (e.g., diversification studies and molecular-clock calibration). The marine snail genus *Alcithoe* exemplifies many of the problems with species identification. The paucity of objective diagnostic characters, prevalence of morphological convergence between species and considerable variability within species that are observed in *Alcithoe* are typical of a broad range of fossilised organisms. Using a synthesis of molecular and morphometric approaches we show that two taxa currently recognised as distinct are morphological variants of a single species. Furthermore, we validate the fossil record for one of these morphotypes by finding a concordance between the palaeontological record and divergence time of the lineage inferred using molecular-clock analysis. This work demonstrates the utility of living species represented in the fossil record as candidates for molecular-clock calibration, as the veracity of fossil species assignment can be more rigorously tested.

## Introduction

Defining species is an age-old problem for evolutionary biologists and reflects, in part at least, the continuous nature of species formation (see [1], [2], [3], [4], [5]). The challenge for palaeontologists is substantial because of the lack, in general, of soft-part anatomy and behavioural characters in fossils, and reliance on hard-part morphology that may itself be incompletely known [6]. Whereas neo-taxonomists *have* successfully used morphological traits to identify distinct units that reflect biological species [7], genetic data have revealed many examples of cryptic or polymorphic species and morphological convergence that demonstrate the shortcomings of morphological species-delimitation [8]. Thus, species as they are understood in the fossil record, do not necessarily equate to species defined in the modern biota [6].

For evolutionary biologists interested in understanding the connection between present and past diversity, the inconsistent use of species concepts is a potential source of error. Thus, for example, morpho-species assignment that fails to distinguish cryptic fossil species could lead to over-estimation of apparent intraspecific genetic variation and rates of molecular evolution, and under-estimation of interspecies diversity and rates of paleontological taxic diversification. Conversely, insufficient sampling of highly variable species (where variability is due to ecophenotypy or sexual dimorphism, for example) could lead to taxonomic over-splitting, inflated estimates of species diversity (e.g. [9]), and under-estimation of molecular evolutionary rates. The fossil record is particularly sensitive to this type of problem due to the existence of incompletely sampled clines in temporal and spatial distributions of species [6]. The costs of misclassification are high when fossil taxa are inferred to be representatives of ancestral clades and used to calibrate nodes above the species level using molecular clocks, leading to erroneous inferences about the age of crown groups and the tempo of species radiation (see e.g. [10], [11], [12], [13], [14], [15]).

With respect to testing of macroevolutionary hypotheses, the general morpho-species problem reduces in part to one of probabilities and frequencies: are morpho-species accurate most of the time or only some of the time? Is there a systematic bias towards over- or under-splitting of species in the fossil record, and does this bias vary from clade to clade, with geological age, or in some other way? On average, for any particular group, is the number of over-split taxa more-or-less balanced by the number of under-split species?

Resolution of the morpho-species problem in both phylogenetic and macroevolutionary contexts requires repeated, combined molecular and morphological analysis and evaluation of individual clades that are sampled widely from the tree of life. From many such analyses a pattern will emerge that will allow biologists and palaeontologists to more accurately assess the strengths and weaknesses of the morpho-species that are used in paleontological inference. The present study is a contribution to this endeavour.

**Figure 1:**
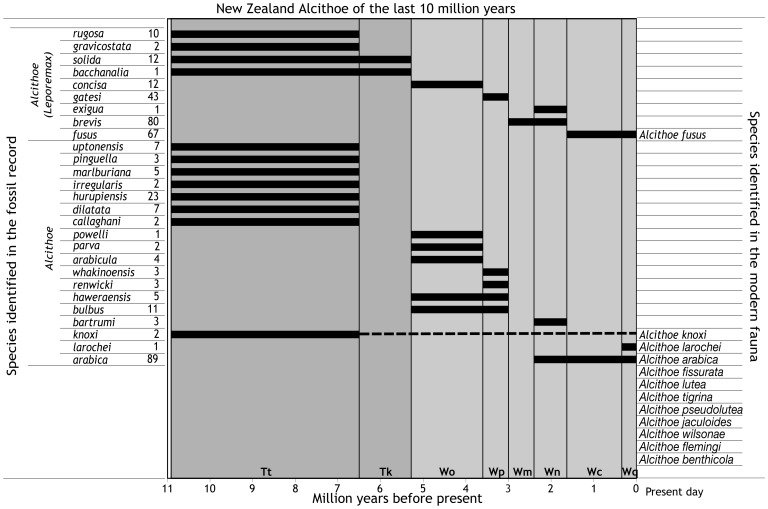
The New Zealand *Alcithoe* of the last 11 million years. Fossil species are shown on the left, extant species on the right. Bars show occurrence in the fossil record in the time bins indicated, with resolution at the level of recognised New Zealand geological stages [56]. Numbers by the fossil species indicate the number of discrete sampling events, as recorded in the FRED database [57]. *Alcithoe knoxi* is known from two collections in Tongaporutuan and is found in the extant fauna, but no fossils have yet been identified from the intervening time, as indicated by a dashed line. Abbreviations are given for New Zealand fossil record stages (Tt – Tongaporutuan, Tk – Kapitean, Wo – Opoitian, Wp – Waipipian, Wm – Mangapanian, Wn – Nukumaruan, Wc – Castlecliffian, Wq – Haweran).

Here we use a clade of marine snail to highlight the problem of molecular-clock calibration when taxonomy is uncertain, the New Zealand endemic genus *Alcithoe*. A rich fossil record and the existence of numerous extant taxa make the genus amenable to molecular-clock analysis (Fig. 1). However, *Alcithoe* taxonomy is problematic because species lack uniquely derived diagnostic morphological characters, are prone to trait convergence and, in some cases, display marked intraspecific morphological variation. As a result, the number of recognised species within *Alcithoe* and their delimitation has altered a number of times, from 15 species and 3 sub-species across 3 genera to 17 species and 8 sub-species in a single genus (e.g. [16], [17], [18]).

**Figure 2:**
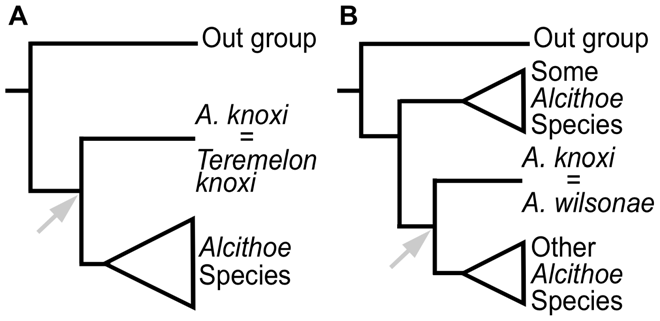
Potential phylogenetic positions of *Alcithoe knoxi*. Morphological ambiguity results in a range of phylogenetic hypotheses for the placement of *A. knoxi*, ranging from (A) *A*. *knoxi* representing a distinct lineage (genus *Teremelon*) sister to the *Alcithoe* lineage, to (B) *A. knoxi* is a form of *A. wilsonae* nested within the modern diversity of *Alcithoe*. The node that would be calibrated at approximately 10my based on the *A. knoxi* fossils is shown (arrows). Calibration (A) would set a maximum limit of the origin of the modern *Alcithoe* at no earlier then 10mya, whereas (B) would allow the diversification of the extant *Alcithoe* to have occurred earlier.

Taxonomic uncertainty due to morphological convergence in shell characters substantially reduces confidence in any assignment of extinct taxa to stem branches in phylogenetic hypotheses for *Alcithoe*, which in turn influences calibration of molecular-clock analysis. Fortunately, three extant *Alcithoe* taxa with consistent and stable species diagnoses are represented in the fossil record: *A. fusus*, *A. arabica* and *A. knoxi*. *Alcithoe fusus* and *A. arabica* are known from extensive palaeontological collections (Fig. 1), so taxonomic assignments of fossils of these species are likely to be robust and thus these fossils can be used with some confidence in clock calibration. In contrast, fossil *A. knoxi* (formerly *Teremelon knoxi*) are known from only two collections of Tongaporutuan age (10.92–6.5 Ma), resulting in a 6.5 Myr gap in the fossil record of this species. Furthermore, as the number of extant *Alcithoe* specimens in collections has increased, morphological overlap between *A. knoxi* and the morphologically variable living *A. wilsonae* has become progressively more apparent [18]. The *A. knoxi* fossils from the Tongaporutuan Stage are important for the calibration of *Alcithoe* molecular-clocks because they potentially represent a deep node in the phylogeny, but only if the fossils and the modern specimens represent the same lineage. There are two primary hypotheses for the phylogenetic position of *A. knoxi* (see Fig. 2). Calibration regimes based on these hypotheses will result in very different estimates of divergence times and rates of molecular evolution.

We examined the phylogenetic position of extant *A. knoxi*, and used mtDNA sequence and independently dated molecular analysis to determine whether the modern and fossil specimens could belong to a single lineage. To do this we needed to establish the species boundaries of extant *A. knoxi* by characterising shell shape of *A. knoxi* and *A. wilsonae* using two-dimensional morphometric methods, and to document genetic variability within and among extant *A. knoxi* and *A. wilsonae*.

## Materials and Methods

### Taxonomic Setting

The marine snail genus *Alcithoe* H. & A. Adams, 1853 is a New Zealand representative of the gastropod family Volutidae Rafinesque, 1815. Extant *Alcithoe* species are found in soft sediments from shallow estuaries to depths of over 1000 m on the continental slope, and Cenozoic fossils of the genus are abundant [19]. The modern fauna consists of 17 species, recognised on morphology, plus several subspecies and forms [18]. *Alcithoe wilsonae* exemplifies the problem of taxonomic instability due to intraspecific morphological variation. In 1979 Powell recognised three living species of *Pachymelon* (*P. wilsonae* Powell, 1933, *P. smithi* Powell, 1950, and *P. grahami* Powell, 1965), and also recognised *Alcithoe* (*Leporemax*) *chathamensis* Dell, 1956 on the basis of geographic distribution and shell morphology. In more recent treatments of *Alcithoe*, Bail and Limpus [18] synonymised all these entities into a single subspecies *A. wilsonae wilsonae*, retaining recognition of the forms *smithi, grahami* and *chathamensis* within the subspecies. In addition, they erected a new sub-species, *A. wilsonae acuminata*, based on shell characters [18]. Powell’s original separation of these various forms as distinct species most likely resulted from relatively sparse sampling and consequent failure to recognise intermediate morphologies, and from the inference that many of these forms appeared to have distinct geographical ranges. Subsequent synonymy was the result of new samples that bridged apparent morphological and geographical discontinuities [18].

**Figure 3:**
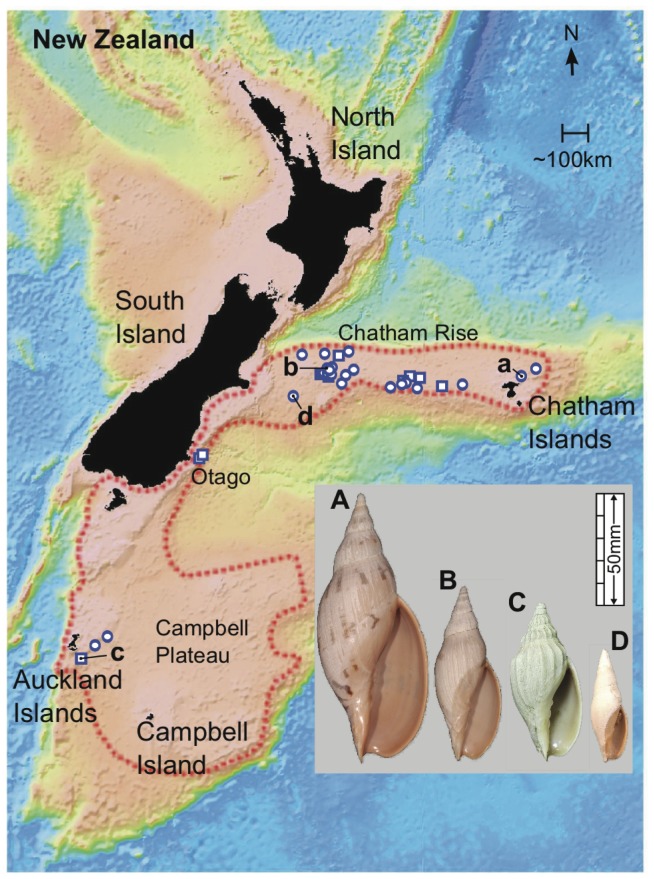
Approximate geographic range of *A. wilsonae* and *A. knoxi*. The theoretical extent of the range of these species is indicated by the dotted line. *A. wilsonae* generally occupies depths from 50 m to 450 m and *A. knoxi* is found between approximately 400 and 750 meters. Approximate locations of sample sites of the DNA sequenced specimens are indicated with circles, boxes indicate the locations of samples only used for morphometric analysis. Inset – A sample of the morphological variation of *A. wilsonae* (A) M190111, (B) M190079, (C) M177177, and the *A. knoxi* form (D) 30037. The sampling locations of these specimens are indicated with the corresponding lower case letter.

In contrast, *Alcithoe knoxi* has been taxonomically stable, although up until 2005 it was retained within *Teremelon*, an otherwise extinct New Zealand genus [18]. *Alcithoe wilsonae* and *A. knoxi* have similar geographic distributions (Fig. 3), but are thought to differ in bathymetric ranges; *A. wilsonae* is typically recorded down to ∼500 m and *A. knoxi* is largely restricted to water depths of ∼450 to 750+ m. As individuals of both species appear sparsely distributed within their ranges, and are from relatively deep water, neither has been extensively sampled.

### Material

Recent sampling trips and commercial fisheries by-catch specimens have increased the number of well-preserved *A. wilsonae* and *A. knoxi* available for morphometric and molecular analysis. Collection locations of the specimens of these two species used in this study are summarised in Table 1. Additional DNA sequence data were obtained for five other *Alcithoe* species: *A. lutea*, *A. fusus*, *A. arabica*, *A. benthicola* and one out-group species, *Amoria hunteri* [20]. All specimens were identified by Bruce Marshall, collection manager of molluscs at the Museum of New Zealand Te Papa Tongarewa, using shell characters (e.g. [17], [18]). Specimens were loaned from Te Papa Tongarewa and the National Institute of Water and Atmospheric Research (NIWA).

**Table 1:** Specimens of *Alcithoe wilsonae* and *A. knoxi* used to determine population variability in mitochondrial DNA (first 35), and shell morphometrics.

Voucher number^a^	GenBank Accessions	Taxon ID	General location	Lat. S^b^	Long. E^b^	Depth (m)
30009/1	JX887177	*wilsonae*	Western Chatham Rise	43.843	176.705	479
30009/2	JX887187	*wilsonae*	Western Chatham Rise	43.843	176.705	479
30034	JX887198	*knoxi*	Western Chatham Rise	44.135	174.844	516–518
30037	JX887179	*knoxi*	Western Chatham Rise	44.135	174.844	516–518
30098/1	JX887192	*wilsonae*	Central Chatham Rise	44.018	178.523	767
30098/2	JX887189	*knoxi*	Central Chatham Rise	44.018	178.523	767
30098/3	JX887197	*wilsonae*	Central Chatham Rise	44.018	178.523	767
30113	JX887188	*knoxi*	Central Chatham Rise	43.873	179.021	480–487
30151/1	JX887193	*wilsonae*	Central Chatham Rise	43.984	179.624	531–532
30151/2	JX887204	*wilsonae*	Central Chatham Rise	43.984	179.624	531–532
30151/3	JX887194	*wilsonae*	Central Chatham Rise	43.984	179.624	531–532
30201/1	JX887185	*wilsonae*	Western Chatham Rise	43.387	175.227	309–310
30201/2	JX887195	*wilsonae*	Western Chatham Rise	43.387	175.227	309–310
30227/1	JX887180	*wilsonae*	Western Chatham Rise	43.469	177.145	251–254
30227/2	JX887186	*wilsonae*	Western Chatham Rise	43.469	177.145	251–254
30227/3	JX887202	*wilsonae*	Western Chatham Rise	43.469	177.145	251–254
30227/4	JX887209	*wilsonae*	Western Chatham Rise	43.469	177.145	251–254
30236	JX887205	*knoxi*	North East of Chatham Islands	43.291	184.438	638–644
30265	JX887199	*wilsonae*	North East of Chatham Islands	43.511	183.824	194–218
30266	JX887184	*knoxi*	North East of Chatham Islands	43.511	183.824	194–218
30297	JX887196	*knoxi*	Central Chatham Rise	43.841	181.411	460–462
M183116	JX887200	*knoxi*	Central Chatham Rise	43.833	179.189	479–486
M190062	JX887206	*wilsonae*	Western Chatham Rise	43.502	176.237	381–385
M190067/1	JX887176	*wilsonae*	Western Chatham Rise	43.570	176.018	350
M190072/1	JX887208	*wilsonae*	Western Chatham Rise	43.570	176.080	360–376
M190079	JX887183	*wilsonae*	Western Chatham Rise	43.500	176.100	360–376
M190088	JX887201	*wilsonae*	Western Chatham Rise	43.602	176.233	354–365
M190092	JX887190	*wilsonae*	Western Chatham Rise	43.483	176.200	380
M190096/1	JX887182	*wilsonae*	Western Chatham Rise	43.633	176.268	377–380
M190101	JX887178	*wilsonae*	Western Chatham Rise	43.468	176.123	339–375
M190111	JX887203	*wilsonae*	North East of Chatham Islands	43.400	–176.202	330–335
M190127	JX887207	*wilsonae*	Western Chatham Rise	43.600	176.133	355–364
M190129	JX887175	*wilsonae*	Western Chatham Rise	43.033	177.017	354–360
M190345	JX887191	*wilsonae*	East of Auckland Islands	50.627	167.437	412–414
M274008	JX887181	*wilsonae*	East of Auckland Islands	50.800	167.017	410
M190096/2	N/A	*wilsonae*	Western Chatham Rise	43.633	176.268	377–380
M190072/2	N/A	*wilsonae*	Western Chatham Rise	43.570	176.080	360–376
M190067/2	N/A	*wilsonae*	Western Chatham Rise	43.570	176.018	350
M116984/A	N/A	*wilsonae*	Western Chatham Rise	43.035	176.650	400
M116984/B	N/A	*wilsonae*	Western Chatham Rise	43.035	176.650	400
M116984/C	N/A	*wilsonae*	Western Chatham Rise	43.035	176.650	400
M116984/D	N/A	*wilsonae*	Western Chatham Rise	43.035	176.650	400
M116984/E	N/A	*wilsonae*	Western Chatham Rise	43.035	176.650	400
M116984/F	N/A	*wilsonae*	Western Chatham Rise	43.035	176.650	400
M116984/G	N/A	*wilsonae*	Western Chatham Rise	43.035	176.650	400
M116984/H	N/A	*wilsonae*	Western Chatham Rise	43.035	176.650	400
M116984/I	N/A	*wilsonae*	Western Chatham Rise	43.035	176.650	400
M116984/J	N/A	*wilsonae*	Western Chatham Rise	43.035	176.650	400
M116984/K	N/A	*wilsonae*	Western Chatham Rise	43.035	176.650	400
M116984/L	N/A	*wilsonae*	Western Chatham Rise	43.035	176.650	400
M119010/A	N/A	*wilsonae*	South of Auckland Islands	51.167	166.617	490–510
M119010/B	N/A	*wilsonae*	South of Auckland Islands	51.167	166.617	490–510
M119010/C	N/A	*wilsonae*	South of Auckland Islands	51.167	166.617	490–510
M119010/D	N/A	*wilsonae*	South of Auckland Islands	51.167	166.617	490–510
M119010/E	N/A	*wilsonae*	South of Auckland Islands	51.167	166.617	490–510
M117117/A	N/A	*wilsonae*	Off Auckland Islands	51.167	166.667	360–390
M117117/B	N/A	*wilsonae*	Off Auckland Islands	51.167	166.667	360–390
M117117/C	N/A	*wilsonae*	Off Auckland Islands	51.167	166.667	360–390
M970087/B	N/A	*wilsonae*	off Otago Peninsula	45.817	171.083	128–179
M970087/C	N/A	*wilsonae*	off Otago Peninsula	45.817	171.083	128–179
M970087/D	N/A	*wilsonae*	off Otago Peninsula	45.817	171.083	128–179
M90027	N/A	*knoxi*	Central Chatham Rise	43.940	180.582	303–296
M10461	N/A	*knoxi*	Central Chatham Rise	43.667	179.467	402
M33600/A	N/A	*knoxi*	Taiaroa Trench, Otago	45.767	171.100	722–768
M183116	N/A	*knoxi*	Central Chatham Rise	43.833	179.189	479–486
M10456/A	N/A	*knoxi*	Central Chatham Rise	43.700	179.917	512
M10456/B	N/A	*knoxi*	Central Chatham Rise	43.700	179.917	512
M10454/B	N/A	*knoxi*	Central Chatham Rise	43.667	179.467	402
M10454/C	N/A	*knoxi*	Central Chatham Rise	43.667	179.467	402
M10454/D	N/A	*knoxi*	Central Chatham Rise	43.667	179.467	402
R4726/B	N/A	*knoxi*	Papanui Canyon, Otago	45.833	171.017	540–590
R4726/C	N/A	*knoxi*	Papanui Canyon, Otago	45.833	171.017	540–590
R4726/D	N/A	*knoxi*	Papanui Canyon, Otago	45.833	171.017	540–590
R4726/E	N/A	*knoxi*	Papanui Canyon, Otago	45.833	171.017	540–590
R4726/F	N/A	*knoxi*	Papanui Canyon, Otago	45.833	171.017	540–590

a voucher numbers with an M prefix denote specimens from the Museum of New Zealand Te Papa Tongarewa collection, voucher numbers with no letter prefix are from the collection of the National Institute of Water and Atmospheric Research (NIWA). Underlined voucher numbers indicate specimens that were not included in morphometric analyses.

b coordinates are given in decimal degrees, rounded to 3 decimal places.

### Morphometric Analysis of Shape

To test the relationship between morphological and molecular classifications we used two, two-dimensional morphometric methods to characterise shell shape in *A. wilsonae* and *A. knoxi*. First, we used Fourier shape analysis of the aperture outline and the approach of Crampton and Haines [21] and Haines and Crampton [22]. Second, we used geometric morphometric analysis (e.g. [23]) of a set of landmarks around the entire profile of the shell in ventral (apertural) view. Results from these analyses were similar and conclusions the same; for this reason we describe and report here results from the geometric morphometric analysis only. As we are not interested here in ontogenetic effects, analysis was restricted to adult or subadult individuals, identified by the presence of a thickened and/or reflected outer lip. For morphometric analysis, 45 specimens of *A. wilsonae* and 18 specimens of *A. knoxi* were considered. Currently known fossil specimens of *A. knoxi* retain suitable traditional morphological characters for identification, but are insufficiently complete to assign all of the landmarks used in our morphometric analysis, therefore this study does not include fossil material.

Geometric morphometric analysis has been applied in a wide range of biological studies (e.g. [23], [24] and references therein). The method uses the spatial distribution of biologically meaningful, morphological landmarks to describe the shapes of organisms, compare distributions of populations of such shapes, and visualize deformations that relate different shapes to each other. Importantly, during analysis, confounding effects of location, scale (size), and rotation are removed during partial Procruses superimposition, so that pure ‘shape’ can be studied in isolation. The approach has strong and well-understood statistical and shape-theoretical underpinnings [25].

**Figure 4:**
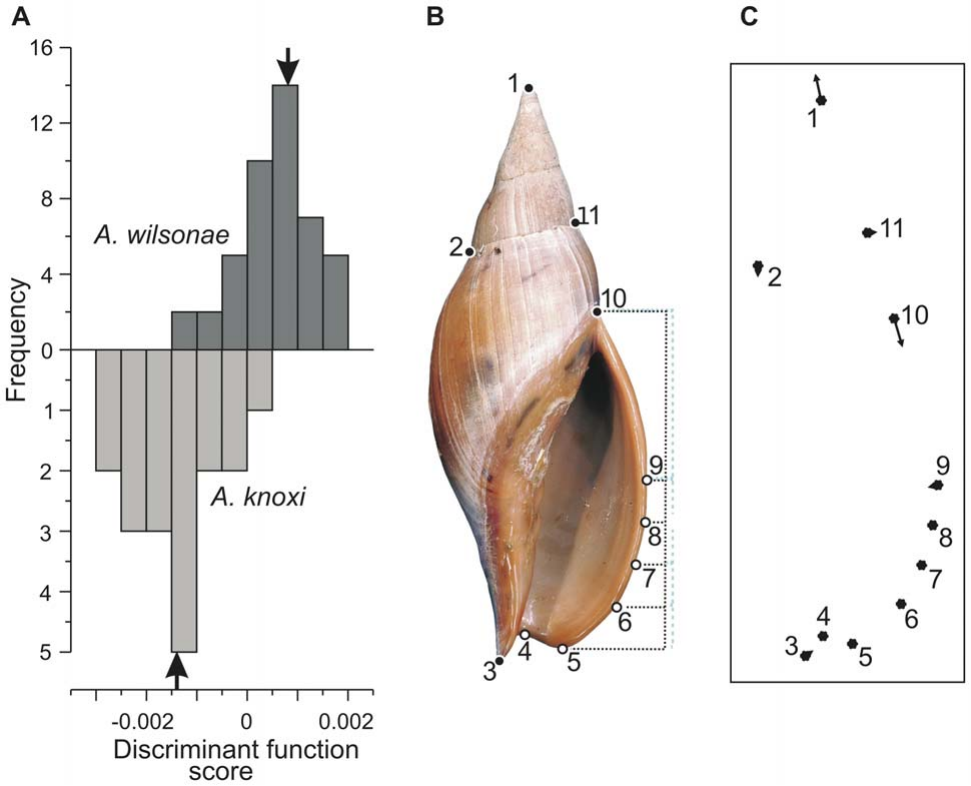
Morphometric analysis of *Alcithoe wilsonae* and *A. knoxi* reveals overlapping distributions, but different means of shell shape. (A) Histograms showing the distribution specimens arrayed according to their scores on the morphometric discriminant function axis. Bold arrows indicate the positions of group means. (B) Landmarks used in the geometric morphometric analysis. Black-filled landmarks are type 1 and type 2 landmarks (*sensu* [26]); white-filled landmarks are semilandmarks (see Table S1 for explanation). The “comb” used to locate semilandmarks 6–9 is shown with a dotted line. This comb was located using the anterior extremity of the outer lip (semilandmark 5) and the suture at the posterior limit of the outer lip (landmark 10), and the anterior half of the comb was divided into eighths in order to locate semilandmarks 6–9. (C) The deformation (i.e. translation of landmarks) inferred from the change from mean *A. wilsonae* to mean *A. knoxi* (right). For clarity, translations have been exaggerated by a factor of two.

In the present study, we used five landmarks (*sensu* [26]) around the shell profile and six semilandmarks around the anterior part of the outer lip of the aperture (Fig. 4, also see Table S1). Inclusion of semilandmarks is a relatively new refinement to the geometric morphometric method, and the concept refers to points that are placed arbitrarily along a curve of interest: the curve is biologically homologous, but the placement of semilandmarks on that curve is not (e.g. [24]). The incorporation of semilandmarks into a study permits analysis of components of shape that lack explicitly defined landmarks. During processing, semilandmarks are allowed to ‘slide’ along the curve in order to minimize variation in shape that is due simply to arbitrary placement of the points. For sliding of semilandmarks we used Procrustes distance, which Perez et al. [27] found was preferred when morphological variation is relatively small. It is important to note that the inclusion of semilandmarks in a geometric morphometric analysis reduces the degrees of freedom in ways that are difficult to quantify. For this reason, statistical analyses based on such data should employ nonparametric bootstrap resampling approaches [23], [24].

The present analysis proceeded via the following steps. Landmark *xy*-coordinates were captured from digital photographs using an A3-size digitizing tablet and stylus, and the software tpsDig v. 2.16 [28]. A ‘comb’ used to locate semilandmarks 6–9 was first pasted into the photographs, translated and resized manually using Adobe Photoshop CS2 v. 9.0.2 (see Fig. 4). Resultant tpsDig data files were manipulated and reformatted using the Integrated Morphometric Package (IMP) program CoordGen6f [23], [29]. Placement of semilandmarks was adjusted to minimize Procrustes distance using the IMP program Semiland6. Data were saved as partial Procrustes coordinates.

Relationships between populations of partial Procrustes coordinates were examined using standard multivariate ordination techniques: Q-mode principal components analysis (PCA) and discriminant function analysis (DFA), implemented in the IMP programs PCAGen6n and CVAGen6j, respectively. Using a function in CVAGen6j, specimens were assigned to one of the two nominal taxa on the basis of distances from group means on the DF axis, and the assignment reliability was determined using a Monte Carlo simulation [30]. Statistical differences between populations of *A. wilsonae* and *A. knoxi* were determined using a bootstrapped *F*-test, as computed by the IMP program TwoGroup6h (900 replications). The scores of specimens on the PC or DF axes formed shape variables used in additional analyses described below.

Relationships between shell shape and water depth were examined using general linear models (GLM), treating shape as the dependent variable and water depth, geographic region and nominal taxonomic group as the independent variables. Results using the first principal component (PC1), which explains 66% of the shape variance, are reported here. This variable was selected because of its apparent relationship with water depth in bivariate plots, and based on the results of exploratory GLM analyses. (We reiterate that, unlike some biometric analyses, PC1 here does not correspond to a size vector, size having been eliminated during the process of partial Procrustes superimposition of outlines.) Geographic region was classified into five areas: eastern, central, and western Chatham Rise, Otago, and the Auckland Islands. Three linear models of increasing complexity were fitted to the data: a simple model in which taxonomic grouping was ignored; a model in which the regression slope is independent of taxonomic grouping, but the intercept is not; and a model with an interaction effect such that slope and intercept depend on taxonomic grouping. The ‘best’ model was selected on the basis of analyses of variance of nested simple-complex models, this is supported by results using an Akaike Information Criterion (AIC) model selection procedure which yielded the same preferred model (results not shown). Because of uncertainties concerning degrees of freedom in a study including semilandmarks, the significance of *F*-statistics used to assess model fit and *t*-statistics used to assess regression coefficients was determined by bootstrapping (1000 replications). During this process, taxonomic group membership and water depth were randomised and two random subsets of data (containing 18 and 45 individuals) were sampled with replacement. The observed *F*-statistics were then compared with the empirical cumulative density function of bootstrapped *F*-statistics to derive probabilities for the observed values. General linear modelling was undertaken using *R* v. 2.12.1 [31].

Sensitivity analysis undertaken as part of a larger study (to be reported elsewhere) has quantified intra- and inter-operator errors associated with orientation of specimens for photography, placement of the comb, and digitization of landmarks. The sensitivity analysis was based on photography and digitization of a single specimen of *A. arabica* by five different operators, and re-photography and re-digitization of the same specimen five times by a single operator. Intra-operator error, quantified as multivariate variance (see [32]), is just 1.5% of the intraspecific variation. Inter-operator error is 2.6% of intraspecific variation. From these results we infer that intra- and inter-operator error is negligible compared to intraspecific shape variation in *Alcithoe* and is unlikely to bias results presented here.

### DNA Extraction, Amplification and Sequencing

Approximately 0.5 mg of tissue was incubated in 300 ml of high-salt buffer with 1 ml of 10 ng/ml ProtK, shaking at 60°C for at least 16 hours. 300 ml of phenol was added and the solution incubated with shaking at room temperature. Following centrifugation, the aqueous phase was removed and mixed with 400 ml of chloroform:isoamyl alcohol (24∶1). The chloroform wash was repeated, and DNA precipitated with 95% ethanol at −20°C for 8 to 16 hours, before centrifugation, washing, drying and resuspension in 0.1 M TE. DNA concentrations were determined using a NanoDrop ND-1000 spectrophotometer (NanoDrop Technologies Inc.). DNA extractions were diluted to approximately 1 ng/ml for amplification.

Fragments of mitochondrial NADH 2 (*nad*2) gene were produced using a primer in the adjacent serine tRNA (NG_mtSER2f: 5′ - AGA AAA AAC TTG GAG TAA ARC AGG GC) paired with a primer in the 5′ end of *nad*2 (NG_mtND2r781∶5′ – CAA AAC CAA GTA AAG GNG GYA ARC C). PCR used Red-Hot Taq (ABgene), following the manufacturer’s instructions with a MgCl_2_ concentration of 2.0 mM. Standard thermal cycling conditions were followed, with 50°C annealing temperatures and 30–35 cycles, in a Biometra^tm^ T1 thermocycler. PCR products were sequenced with both forward and reverse primers using BigDye Terminator v3.1 and sequenced with an ABI 3730.

### Population Genetic Analysis

DNA sequences of up to 823 base pairs were amplified from the mitochondrial NADH 2 (*nad*2) gene. Sequences were edited and aligned using Sequencher (v4.6, Gene Codes Corporation, Ann Arbor, Michigan). Alignments were trimmed to the start codon of *nad*2 on one end and trimmed flush on the other. The resulting alignment was translated in SE-AL v2.0a11 [33] to confirm that nucleotide sequences did not contain erroneous stop codons that would be indicative of nuclear coded mitochondrial pseudogenes. As three samples did not sequence to the *nad*2 start codon, the alignment was further trimmed to remove sites containing missing data from these samples for analysis. The final alignment was 573 bp in length.

Trees were inferred from the nucleotide alignment using Neighbor-Joining and Maximum Likelihood approaches, as implemented in Geneious v4.7.5 [34]. Best-fit nucleotide substitution models were found using Modeltest [35] as implemented in HYPHY v1 [36]. A haplotype network for the *A. wilsonae* and *A. knoxi* samples was constructed using parsimony. Analysis of molecular variance (AMOVA) used GENEALEX 6 [37]. The total sample of 35 snails was partitioned into subgroups according to three criteria: 1. Current taxonomy: *A. knoxi* (n = 8), *A. wilsonae* (n = 27). 2. Geographic location: Western Chatham rise (n = 20), Central Chatham rise (n = 9), North East of the Chatham Islands (n = 4), East of the Auckland Islands (n = 2). 3. Bathymetry: less than 450 m (n = 21), 450 to 500 m (n = 7), deeper than 500 m (n = 7).

### Dated Analysis

Molecular-clock analyses used BEAST v1.4.8 [38], to estimate the divergence time of the lineage leading to *Alcithoe wilsonae*. The taxa included in the molecular-clock analysis were selected to maximise the number of nodes that could be calibrated with fossil data, and to minimise topological inconsistencies associated with low phylogenetic resolution at deeper nodes due to the use of the short *nad*2 DNA fragment available for the *A. wilsonae* and *A. knoxi* samples. The taxon set, taken from Hills *et al.* [20], comprised 2 out-group taxa and 11 *Alcithoe* species, including the *Alcithoe wilsonae* specimen M190062 (see Table 1). The molecular data consisted of the mitochondrial gene set recommended by the Hills *et al.* [20] analysis (nad2, cox1, atp6 and 16S), a total alignment of 4696 bp.

To avoid circularity, the analysis was calibrated using palaeontological data for *A. arabica* and *A. fusus*. Calibration of the root node of the tree was based on three observations of the paleontological record of the New Zealand volutes. First, the earliest fossil volute known from New Zealand from the Teurian stage, 65–55 Ma [19]. Second, the next oldest record occurs in the Bortonian stage (43–37 Ma) [19], so there is some uncertainty as to the age of the New Zealand volute lineage. Third, that the isolation of the New Zealand volutes from the Australian fauna was established by approximately the same time as the Bortonian fossils in the mid-Tertiary [39]. Based on this paleontological data root node was calibrated using a lognormal prior distribution with a lognormal mean of 2, standard deviation of 0.4 and the distribution was offset along the x axis by 43 million years. This distribution placed 95% of the prior probability of the root node between 46.4 Ma and 59.2 Ma, which reflects the conservative interpretation that the New Zealand volute lineage leading to *Alicthoe* was established by the beginning of the Bortonian stage and is unlikely to have predated the Teurian fossils. Two internal nodes were calibrated with fossil data: the node representing divergence of *A. arabica* and the node representing divergence of *A. fusus*. Both these species have been sampled extensively in the fossil record (see Figure 1), and therefore earliest occurrences are considered reasonable estimates for their times of origination. The earliest recognised fossil of *A. arabica* is from the Nukumaruan Stage (2.4–1.63 Ma) of the New Zealand geological timescale, and the earliest fossil of *A. fusus* is from the Castlecliffian Stage (1.63–0.34 Ma) [19]. These nodes were each calibrated with lognormal distributions where the 2.5% quantiles were set as close to the younger bound of the relevant time interval as possible, and the majority of the 95% highest probability density interval (HPD) was distributed between this and the older bound (*A. arabica* node: mean  = 0.7, SD  = 0.12; *A. fusus* node: mean  = 0.01, SD  = 0.38, exponentially transformed offset = −0.2). A Yule (pure birth) prior was used as this analysis involves predominantly interspecific relationships. A GTR+I+G nucleotide substitution model was applied, as inferred for the dataset by ModelTest. The MCMC chain was run for 10 million generations, sampling every 1000. Input XML files were generated using BEAUTi v1.4.8. Output log files from BEAST were inspected in Tracer v1.4.1 to confirm stationarity of sampling traces and summary statistics with adequate effective sample sizes. The maximum credibility tree was generated from the resulting sample using TreeAnnotator v1.4.8, and visualised in FigTree v1.2.3.

## Results

### Morphometric Analysis of Shape

Populations of *A. wilsonae* and *A. knoxi* occupy overlapping regions of morphospace, as shown by both discriminant function (Fig. 4) and principal components analyses (results not shown). Means of the two groups are, however, significantly different based on a bootstrapped *F*-test (partial Procrustes distance between means  = 0.0372, *F*  = 16.02, *p*  = 0.0011). Furthermore, an assignments test achieved 89% correct assignment of specimens to their *a priori* nominal groups. Deformation implied by the transition from mean *A. wilsonae* to mean *A. knoxi* corresponds to relative movement of landmark 1 towards the posterior and landmark 10 towards the anterior (Fig. 3). In other words, compared to average *A. wilsonae*, average *A. knoxi* has increased spire height and decreased aperture height.

**Figure 5:**
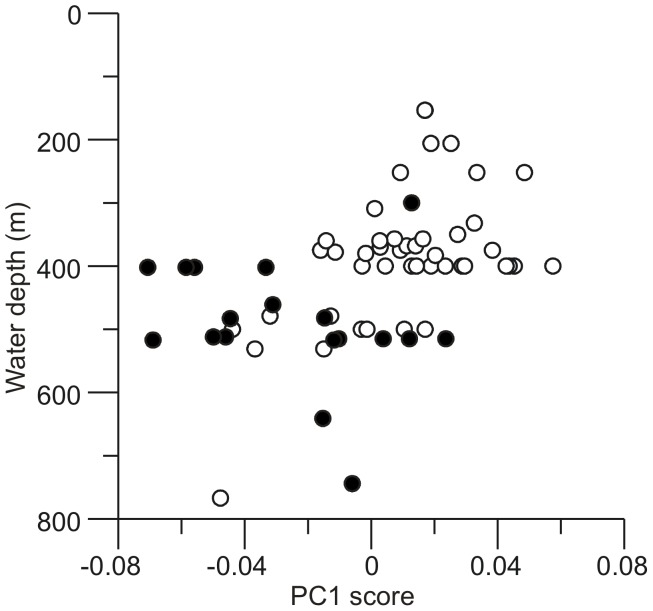
Comparison of morphometric shape with depth of collection of two benthic snail taxa, *Alcithoe wilsonae* and *A. knoxi.* Scores of specimens on the morphometric first principal component axis plotted against water depth indicates a correlation between depth and morphology. The first principal component explains 66% of the total shape variation in the dataset. Open circles represent *A. wilsonae* specimens, filled circles represent *A. knoxi* specimens.

There are indications that morphology may be related to water depth at which the animal lived, given the differences noted above and the fact that *A. knoxi* specimens tend to be taken from deeper water than *A. wilsonae*. To test this we examined the linear relationship between the first principal component (PC1) from the geometric morphometric analysis, which explains 66% of the total shape variation, and water depth. Other principle components were not considered as they each explained less than 10% of the shape variation, reveal little difference between the nominal taxa, and show no apparent relationship with depth. Results from general linear modelling indicate that a model with differing regressions for the two nominal groups is preferred (Table 2), as implied also by visual inspection of the bivariate scatterplot (Fig. 5). This model yields a statistically significant slope relating PC1 to water depth for the *A. wilsonae* group (model *F*  = 15.19, model *p*<0.0001, regression slope = −1.164×10^−4^, *t* = −3.597, *p* = 0.0040); in contrast, the slope term for the *A. knoxi* group is not statistically different from 0 (slope  = 6.971×10^−5^, *t*  = 1.204, *p* = 0.1550). Regressions in which discriminant function scores were used instead of PC1 scores yield similar conclusions (results not shown). We note that, for the *A. wilsonae* group, the inclusion of geographic region in the analysis adds no explanatory power, and this factor was not included in the models reported in Table 2 (in fact, an apparent relationship between shape and geographic region is explained entirely by regional variations in water depth). In the case of *A. knoxi*, there may be some geographic variation in PC1 scores (results not shown), although this has no apparent influence on the relationship between shape and water depth and, further, sample sizes are too small to demonstrate geographic variation unequivocally.

**Table 2:** Results of general linear modelling of the regression between shell shape and water depth.

Model	Simple	Parallel
Parallel	*F* = 14.415, *p* = 0.0010	
Non-parallel	*F* = 11.968, *p*<0.0001	*F* = 7.870, *p* = 0.0070

Three models were tested: simple, regression independent of nominal taxonomic grouping; parallel, regression slope is independent of taxonomic grouping, but the intercept is not; non-parallel, slope and intercept depend on taxonomic grouping. The *p*-values on *F*-statistics indicate the probability that the simpler model in each comparison is adequate; significant *p*-values indicate that the more complex model is preferred (see text). In each comparison the more complex model is supported and, overall, the non-parallel model is preferred, indicating a correlation between depth and morphology.

### Sequence Data

An alignment of 573 bp of nucleotide sequence data from the mitochondrial *nad*2 gene was generated for 27 *A. wilsonae* and 8 *A. knoxi* specimens from 24 trawling locations. These DNA sequences resolved 25 haplotypes, based on 27 variable nucleotide positions. Pair-wise comparisons of sequence dis-similarity within the sample reached a maximum of 0.87% (average of 0.33%), with a high proportion of the observed variability unique to individual haplotypes. In all cases, where more than one individual was sequenced from the same trawl, multiple haplotypes were detected (Table 1). Most haplotypes (18) were found in just one individual; fewer (7) were shared among locations; and only one was sampled more than once from a single dredging site. The greatest geographic distance between samples with identical haplotypes was ∼435 km (between M190067 and 30297; Table 1).

**Figure 6:**
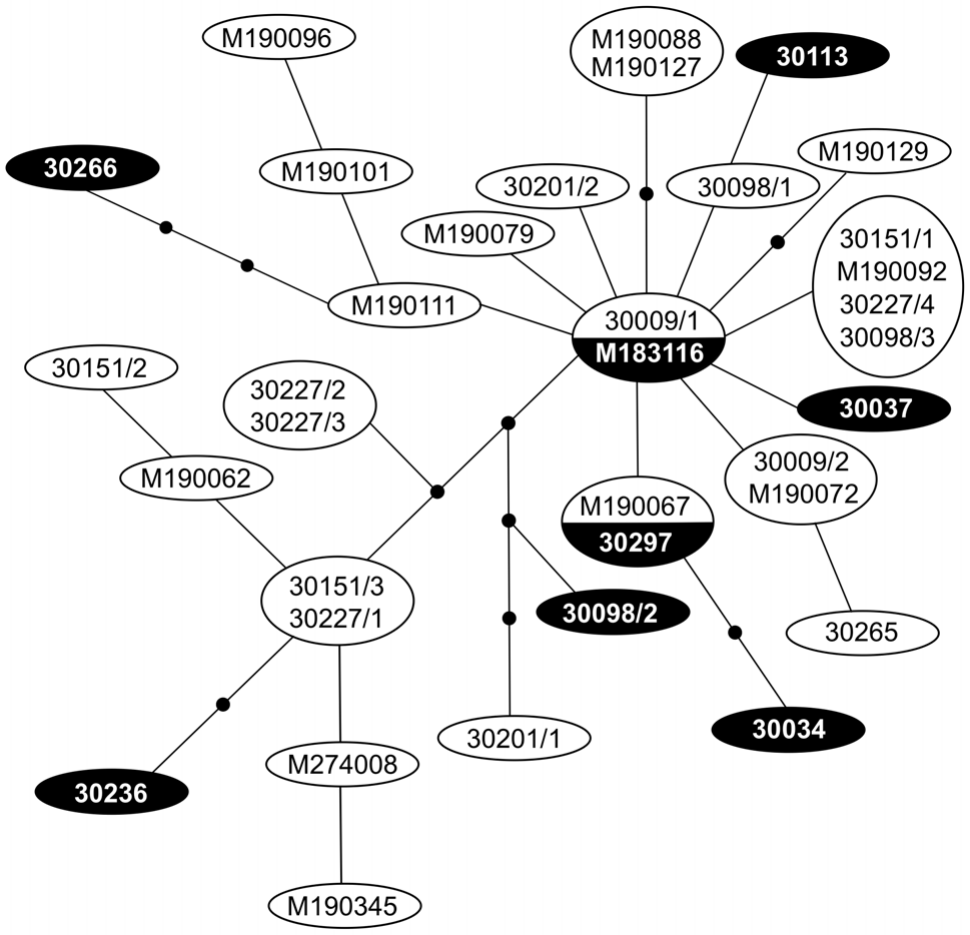
Haplotype network (based on 573 bp of mtDNA *nad*2) of 27 *Alcithoe wilsonae* (open ovals) and 8 *Alcithoe knoxi* (filled ovals) demonstrates the lack of genetic structure related to morphology. Each haplotype differs by a single nucleotide, unsampled haplotypes are represented by black dots. (See Table 1 for sample codes.).

*Alcithoe wilsonae* and *A. knoxi* did not form reciprocally monophyletic groups in phylogenies that included five other members of the genus, nor were any groupings observed that were consistent with nominal *A. wilsonae* sub-species (data not shown). A haplotype network, constructed using the 25 unique haplotypes of *Alcithoe wilsonae* and *A. knoxi* (Fig. 6), reveals no structure concordant with morpho-species. There were no clear patterns indicative of spatial population subdivision. A central haplotype from which other haplotypes radiate (found in both *A. wilsonae* (30009/1) and *A. knoxi* (M183116)), is no more common than other haplotypes as would be expected from recent population growth [40], [41].

### Population Genetic Structure

Snails were assigned to groups based on three criteria; current taxonomy, geographic sampling location, and bathymetry. We found little or no correlation between groupings by any of these criteria and mtDNA haplotype analysis using AMOVA (Table 3). Current taxonomic groupings explained only 1% of the genetic variation, whereas broad geographic population assignments explained 9%. Bathymetry explained only 5% of the genetic variation. *Alcithoe knoxi* specimens were genetically indistinguishable from the *A. wilsonae* specimens (Fig. 6). We infer from the sequence data that these two taxa represent a single biological species with a current or recently large population and find no evidence of barriers to gene flow among morphs or geographic areas. Resampling simulations suggest that the full genetic diversity of *A. wilsonae/A. knoxi* has yet to be sampled and therefore it would be premature to estimate population size from these data.

**Table 3:** Results from AMOVA of *A. wilsonae* nad2 DNA sequneces, low Phi_PT_ values indicate high levels of genetic exchange between populations.

Source of variation	df	Sum of squares	Est. Var.	Percentage variation	PhiPT	P
**Taxonomic Groupings**						
Among Pops	1	1.930	0.011	1%		
Within Pops	33	59.042	1.789	99%		
Total	34	60.971	1.801			
Fixation index					0.006	0.330
**Geographic groupings**						
Among Pops	3	8.579	0.175	9%		
Within Pops	31	52.393	1.690	91%		
Total	34	60.971	1.866			
Fixation index					0.094	0.131
**Grouping by Depth**						
Among Pops	2	5.448	0.101	5%		
Within Pops	32	55.524	1.735	95%		
Total	34	60.971	1.836			
Fixation index					0.055	0.099

### Divergence time of Alcithoe Wilsonae

**Figure 7:**
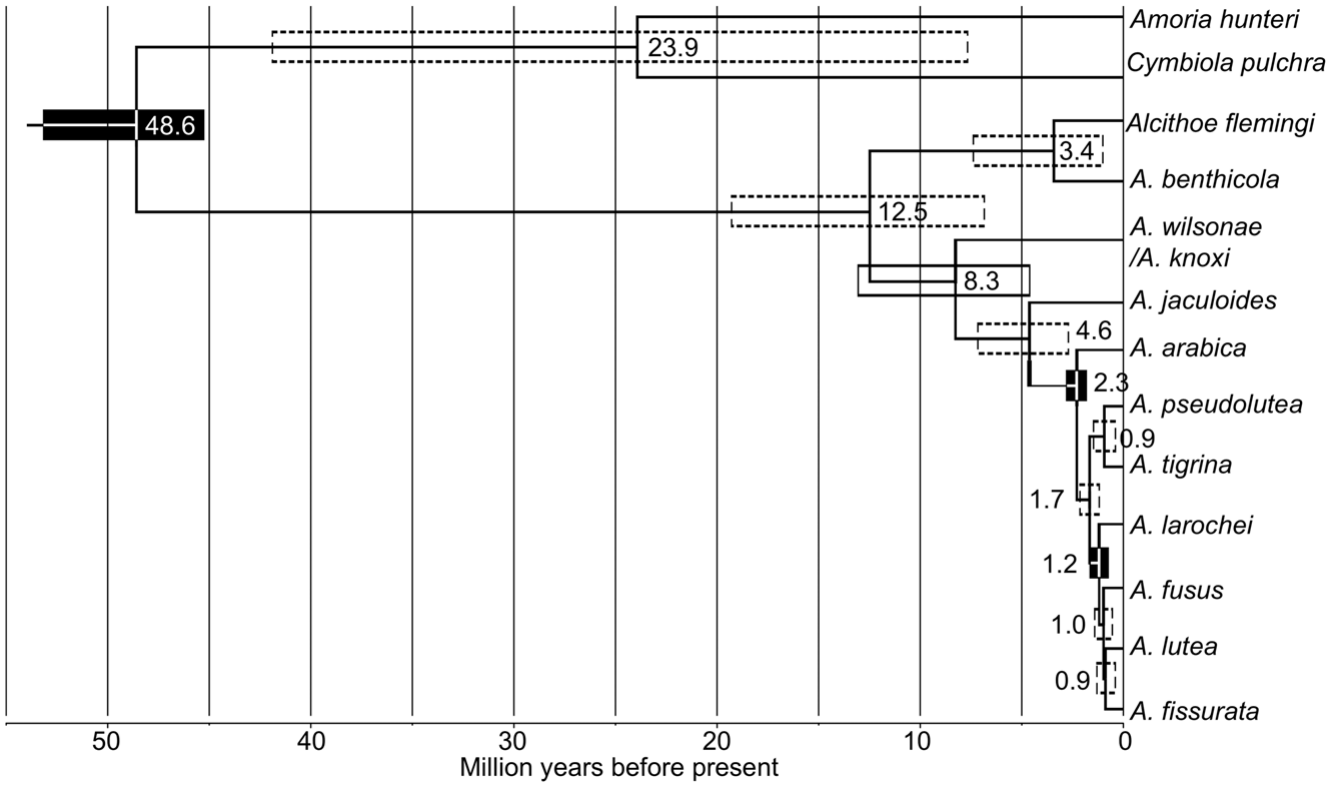
Molecular-clock analysis of the *Alcithoe* phylogeny derived from 4696 **bp of mtDNA sequence indicates that** ***A. wilsonae/knoxi*** **diverged from the** ***A. arabica*** **lineage approximately 8 million years ago.** All nodes are recovered with a posterior support of at least 0.93 and had >90% bootstrap support under Maximum likelihood, with the exception of the *A lutea*/*A. fissurata* node (0.58). Node age estimates are given in millions of years before present. 95% confidence intervals are depicted by boxes centred on nodes. Nodes calibrated using soft priors based on fossil data are indicated with filled boxes.

**Figure 8:**
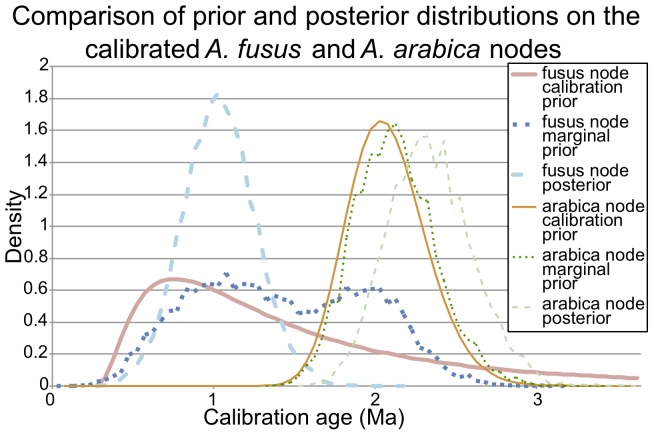
Comparison of prior and posterior distributions on the calibrated *Alcithoe fusus* and *A. arabica* nodes illustrates an interaction between the two node calibrations, but this interaction has little effect with the addition of sequence data. The distribution for the marginal prior on the *A. fusus* node recovers a bimodal distribution that appears to result from leakage into the *A. arabica* node calibration. However, the posterior distribution on the *A. fusus* node shows that the anomalous secondary peak in the marginal distribution has little effect on the analysis in the presence of sequence data.

Molecular-clock analysis, calibrated independently of *A. knoxi*, resulted in an estimated divergence of the *A. wilsonae/A. knoxi* lineage from other *Alcithoe* at between 4.6 and 13.1 million years ago (Fig. 7). High Bayesian posterior support for most nodes indicates that there is little topological uncertainty in the inferred phylogeny. Maximum likelihood analysis confirmed this topological stability, with bootstrap support values of greater than 90 for all branches except for the divergence of *A. fusus* and *A. larochei,* which suffers from a lack of resolution [20]. In order to ensure that the analysis was not adversely affected by artefacts of the calibration regime [42], the distributions of the node calibration priors, the marginal prior (the derived product of all priors applied in the analysis) and the node posteriors were compared (Fig. 8). The marginal prior and the node calibration prior for the *A. arabica* node were almost identical. However, an anomalous bimodal distribution was evident in the marginal prior for the *A. fusus* node, most likely a result of an interaction with the *A. arabica* node prior calibration. However, this bimodal marginal prior appears to have had little effect on the analysis, as the posterior distribution for the *A. fusus* node is unaffected by the anomalous second peak and appears to be consistent with the prior calibration. The inferred divergence time of the *A. wilsonae/A. knoxi* is consistent with the first appearance of *A. knoxi* in the fossil record in the Tongaporutuan stage (Fig. 1). Thus independent calibration supports the species identification of the fossils, and the longevity of *A. knoxi*.

## Discussion

Both interspecific convergence and intraspecific variation of morphology can lead to incorrect assignment of species by palaeo- and neo-taxonomists. Here we have shown how the application of a little genetic data in combination with morphometric analysis enhances interpretation of the taxonomic status of an extremely problematic lineage. We have identified a single morphologically variable species where taxonomists saw two species (and even two genera), and using independent calibration points found molecular support for identifications of 11 – 6.5 million year old fossils.

Population-genetic and morphometric analysis of *Alcithoe wilsonae* and *Alcithoe knoxi* individuals demonstrates that these reflect morphological variation within one species. Sharing of identical haplotypes at a single locus is not in itself conclusive proof of a single species, but in concert with detailed shell shape analysis it provides compelling evidence. Morphometric analysis of shell shape shows that, whereas the group means of the two nominal taxa are significantly different, there is also significant morphological overlap. Therefore, we advocate that *Alcithoe knoxi* ( =  *Teremelon knoxi*) (Dell, 1956) be regarded as a synonym of *Alcithoe wilsonae* (Powell, 1933). The lack of any genetic structure within *Alcithoe wilsonae/knoxi* indicates that a single large population was sampled, with no evident barriers to gene flow. Extensive gene flow apparently occurs at some stage of their life cycle, contrary to expectation for a non-broadcast spawning, direct developing, benthic gastropod.

The lack of an existing fossil record for *A. wilsonae* could suggest that traditional taxonomy identified a very recent incipient speciation, with *A. wilsonae* arising from within *A. knoxi*. Mitochondrial haplotype data would not necessarily have sufficient resolution to characterise this due to incomplete lineage sorting and continuing gene flow. However, it is expected that a reconsideration of the currently recognised fossil species of *Alcithoe* will reveal that one or more are synonyms of *A. wilsonae*. Given the previous instability of the taxonomic status of *A. wilsonae* it would be unsurprising if some fossil specimens have been recognised as separate species. Furthermore, a recent emergence of *A. wilsonae* from within *A. knoxi* would have no bearing on the utility of the *A. knoxi* fossils to calibrate the lineage in molecular-clock analysis.

Morphometric analysis revealed a clear relationship between water depth and morphology. In particular, *wilsonae* specimens exhibit a grade in morphology towards ‘*knoxi*’-like shape with increasing water depth. No morphological grade is observed for ‘*knoxi’* specimens, presumably because this form represents the limit of the species phenotypic response to water depth. Depth related morphological clines have been shown in other deeper-water marine gastropods (e.g. [43]). With increasing depth hydrostatic pressure increases, temperature decreases and is more stable, and food sources are likely to be scarcer [43]. These conditions influence the dynamics of a trade-off between energy availability and the cost of building the shell, which likely leads to differential growth rates giving rise to different shell characteristics. The combined observations of no genetic differentiation between forms, and a morphological gradient from shallow-water to deep-water forms, are consistent with an ecologically determined morphocline in *A. wilsonae*.

The significant difference between the mean shapes of *‘knoxi’* and *wilsonae* illustrates why, particularly when few specimens were available, these two forms have previously been considered separate species. Using traditional discrete morphological characters, separation of the two species has been maintained largely based on specific protoconch and aperture characteristics [18]. One possible explanation for the large relative protoconch size, traditionally one of the primary characters used to distinguish *A. ‘knoxi*’, is an ecological response of embryos or juveniles in deep-water habitat to consume siblings while in the egg capsule (B. Marshall, pers. com.). This behaviour, known as adelphophagy, has been described in other neogastropod species (e.g. [44], [45]), and leads to the generation of a larger protoconch in the surviving offspring due to an increased growth rate during early developmental stages.

Molecular-clock analysis reveals that the time of divergence of the lineage leading to modern *Alcithoe wilsonae* is consistent with the fossil record for *Alcithoe (Teremelon) ‘knoxi*’. Although the median life span of New Zealand Cenozoic marine mollusc gastropod species in the fossil record has been estimated at about 3.1 million years [46], the range of species longevities for marine molluscs is <1 million years up to 30 million years, and 67 described species (∼4%) have ranges of over 10 million years (unpublished result based on dataset of [46]). Thus the approximate 10 million year history for *Alcithoe wilsonae* is not implausible. What is unusual is that we have good fossil and population genetic data for the same taxa. In this lineage extant species represented in the fossil record allow calibration points at both shallow nodes (Pleistocene) and deeper nodes (Miocene).

Our analyses provide compelling evidence that the palaeontological record of specimens previously recognised as *A. ‘knoxi*’ can be accurately applied to an internal node of the *Alcithoe* genus phylogeny (the divergence of *A. wilsonae*) for the purposes of molecular-clock calibration. This is consistent with one of our morphologically based hypotheses (Fig. 2B), and ensures that the calibration will be appropriately applied to the phylogeny. For example, if the palaeontological data were applied differently (e.g. Fig. 2A), then the divergence times of all branches before *A. wilsonae* would be underestimated. Such an underestimation in divergence times would then lead to an overestimation of rates of molecular evolution in these branches.

Molecular studies have sometimes found general concordance between snail species identified by shell morphology and genetic identification (e.g. [47], [48], [49]). Gastropod evolution, however, is notoriously rich in examples of morphological convergence (e.g. [50], [51], [52]). Fossil data are routinely used to estimate changes in biodiversity, species longevity and geographic occupancy of species and these are all topics that could be undermined by failure to accurately diagnose species [53], [54]. For this reason such studies often consider only taxonomic categories above the species level (e.g. [6]), even though processes of speciation and extinction operate at the species level and this would seem to be the appropriate level for analysis [55]. Furthermore, accurate inference of molecular-clocks depends on the correct assignment of fossil calibration data. Using a calibration that is founded on incorrect assumptions about evolutionary relationships (via morphology) could invalidate conclusions. Using extant species with varying life spans in the fossil record removes the problem of where to place “stem” fossils into a phylogeny. With *Alcithoe* we have shown the advantage of using living species with a fossil history to calibrate molecular-clock analysis, as we show that fossil assignments can be tested and independent support for the calibration can be demonstrated.

## Supporting Information

Table S1 Landmarks used in this analysis, classified as types 1–3 according to Bookstein [26] or as semilandmarks (e.g. Sheets [58]).
(DOCX)
