# Supplementary material for: DNA and Morphology Unite Two Species and 10 Million Year Old Fossils
Source: PLoS One. 2012 Dec 20;7(12):e52083. doi: 10.1371/journal.pone.0052083 (PMC3527379; doi:10.1371/journal.pone.0052083)
Supplement: Table S1 — Landmarks used in this analysis, classified as types 1–3 according to Bookstein [26] or as semilandmarks (e.g. Sheets [58]). (DOCX) [file pone.0052083.s001.docx]

**Table S1** —Landmarks used in this analysis, classified as types 1 – 3 according to Bookstein [[1](#_ENREF_1)] or as semilandmarks (e.g. Sheets [[2](#_ENREF_2)]).

| **Landmark** | **Type** | **Description** | **Notes** |
| --- | --- | --- | --- |
| 1 | 1 | Protoconch – i.e., position of larval shell |  |
| 2 | 1 | Left side suture of body whorl |  |
| 3 | 2 | Anterior tip of columella | In optimum presentation, this landmark is at the intersection of the anterior extremity of the fasciole and the inner lip callus; in reality, this point is prone to abrasion, breakage and overgrowth of callus and in some shells its position has been estimated with small uncertainty |
| 4 | semi | Posterior, visible extremity of siphonal notch | The depth of the siphonal notch is an important taxonomic character in the Volutidae and the true posterior extremity of the notch is a type 2 landmark. The point is, however, not co-planar with other points (and thus subject to parallax errors) and in many cases it is obscured by the inner lip on the columella. Furthermore, in some individuals the notch is barely developed. For these reasons we have located landmark 4 at the posterior-most, visible point on the notch or at the corresponding maximum in curvature on specimens lacking an obvious notch, and have processed the point as a semilandmark |
| 5 | semi | Anterior extremity of outer lip | A type 3 landmark but processed here, conservatively, as a semilandmark (see text) |
| 6 | semi | Anterior half of outer lip, located using ‘comb’ | Semilandmarks restricted to anterior part of lip so as to avoid large knobs that affect posterior part of outer lip in some taxa. (This issue is relevant to a larger analysis of Volutidae, of which this study forms part. In any individual, expression of these knobs is determined arbitrarily by the vagaries of adult growth and is not a reliable indicator of adult morphology.) |
| 7 | semi | As above | As above |
| 8 | semi | As above | As above |
| 9 | semi | As above | As above |
| 10 | 1 | Suture at posterior limit of outer lip |  |
| 11 | 1 | Right side suture of body whorl |  |

1. Bookstein FL (1991) Morphometric tools for landmark data; geometry and biology. Cambridge: Cambridge University Press.

2. Sheets HD, Covino KM, Panasiewicz JM, Morris SR (2006) Comparison of geometric morphometric outline methods in the discrimination of age-related differences in feather shape. Frontiers in zoology 3:15.
